# A Case of Open Cholecystectomy for Acute Cholecystitis in a Patient with an Implanted Left Ventricular Assist Device on the Waiting List for a Deceased Donor Heart Transplantation

**DOI:** 10.70352/scrj.cr.25-0812

**Published:** 2026-07-25

**Authors:** Kazushige Migita, Hajime Imamura, Yuki Nunoshita, Kouki Kurotaki, Shun Nakamura, Shinichiro Ogawa, Baglan Askeyev, Ayaka Satoh, Takashi Hamada, Hajime Matsushima, Ayaka Kinoshita, Akihiko Soyama, Tomohiko Adachi, Susumu Eguchi

**Affiliations:** 1 Division of Abdominal and Transplant/Regenerative Surgery, Department of Surgery, Nagasaki University Graduate School of Biomedical Sciences, Nagasaki, Nagasaki, Japan; 2 Department of Innovative Surgical Technology, Nagasaki University Graduate School of Biomedical Sciences, Nagasaki, Nagasaki, Japan

**Keywords:** left ventricular assist device, acute cholecystitis, open cholecystectomy, heart transplantation, percutaneous transhepatic gallbladder drainage

## Abstract

**INTRODUCTION:**

Left ventricular assist devices (LVADs) are increasingly used as bridge-to-transplant or destination therapy for patients with advanced heart failure. As long-term survival improves, more recipients encounter noncardiac surgical conditions. Acute cholecystitis is one of the most common abdominal emergencies in this population and presents unique challenges due to continuous anticoagulation, altered hemodynamic physiology under continuous-flow support, and the presence of pump hardware and drivelines. Delay in treatment risks systemic infection or device contamination, potentially jeopardizing heart transplant eligibility. However, evidence guiding optimal surgical management in patients with prolonged LVAD support remains limited.

**CASE PRESENTATION:**

A man in his 50s with a 6-year history of HeartMate II (Abbott Laboratories, Abbott Park, IL, USA) support presented with fever and right upper quadrant abdominal pain while waiting for deceased donor heart transplantation. Imaging confirmed acute cholecystitis with gallstones. Because of the physiological risks associated with pneumoperitoneum and the need for ongoing anticoagulation, percutaneous transhepatic gallbladder drainage (PTGBD) was performed first, leading to effective infection control. As prolonged catheter placement was undesirable in a transplant candidate, he subsequently underwent elective open cholecystectomy after multidisciplinary planning, perioperative anticoagulation adjustment with heparin bridging, and preoperative US mapping of the driveline. The operation was completed without device injury, uncontrollable bleeding, or hemodynamic instability. The postoperative course was uneventful, and the patient successfully underwent heart transplantation 4 months later.

**CONCLUSIONS:**

This case demonstrates that a staged strategy consisting of early infection control followed by elective cholecystectomy can be safely applied in LVAD-supported patients awaiting heart transplantation. Individualized perioperative planning, optimized anticoagulation management, and precise assessment of driveline anatomy are essential to ensure surgical safety and to preserve transplant eligibility in this high-risk population.

## Abbreviations

LVAD
: left ventricular assist device
PTGBD
: percutaneous transhepatic gallbladder drainage

## INTRODUCTION

LVADs have become an established therapeutic option for patients with end-stage heart failure, serving as either a bridge to heart transplantation or as destination therapy for those who are not transplant candidates.^1)^ With continuous technological improvements in device durability and advances in perioperative management, long-term survival of LVAD recipients has markedly improved. Consequently, an increasing number of patients living with durable LVAD support are now encountering noncardiac surgical conditions during the course of their treatment.^2)^

Among these conditions, acute cholecystitis represents a particularly important clinical scenario because it carries a substantial risk of systemic infection, potential contamination of the driveline or pump pocket, and consequent loss of eligibility for heart transplantation. Management of acute cholecystitis in patients with LVAD support is therefore uniquely complex. Continuous-flow physiology renders these patients highly preload-dependent and susceptible to hemodynamic instability under general anesthesia or pneumoperitoneum. In addition, mandatory long-term anticoagulation to prevent pump thrombosis creates a delicate balance between bleeding and thromboembolic risks in both emergent and elective surgical settings.^2)^

For high-risk LVAD patients presenting with biliary infection, percutaneous gallbladder drainage is often selected as the initial management strategy to achieve rapid infection control with minimal physiological stress. However, definitive cholecystectomy is frequently required to prevent recurrent infection, avoid catheter-related complications, and maintain transplant candidacy.^3)^ Despite the growing clinical need, evidence regarding optimal operative timing, perioperative anticoagulation management, and the choice of surgical approach in this patient population remains limited, particularly among transplant candidates requiring prolonged LVAD support.

Here, we report a case of acute cholecystitis in a patient with long-term LVAD support who was awaiting heart transplantation, successfully managed with a staged strategy consisting of PTGBD followed by elective open cholecystectomy. This case emphasizes the importance of multidisciplinary planning, tailored anesthetic and anticoagulation strategies, and meticulous preoperative assessment to safely treat biliary disease in this complex and high-risk population.

## CASE PRESENTATION

A man in his 50s with a long-standing history of dilated cardiomyopathy and advanced chronic heart failure had undergone implantation of a continuous-flow HeartMate II LVAD (Abbott Laboratories, Abbott Park, IL, USA) 6 years earlier as bridge-to-transplant therapy. Since implantation, he had remained on the heart transplant waiting list and required lifelong anticoagulation with warfarin to prevent thromboembolic complications related to the device. He had also been receiving aspirin 100 mg/day as antiplatelet therapy. Aspirin was temporarily withheld perioperatively and was restarted at 100 mg/day on POD 2 after confirming the absence of active bleeding.

The patient presented with right upper quadrant abdominal pain and fever that began 9 h before admission. On arrival, vital signs were as follows: temperature 36.2°C, blood pressure 122/62 mmHg, heart rate 75 bpm, and SpO_2_ 98% on room air. Abdominal examination revealed Murphy’s sign (−) without guarding. Laboratory tests showed white blood cell count 11.9 × 10^3^/µL, hemoglobin 9.7 g/dL, platelet count 138 × 10^3^/µL, C-reactive protein 5.88 mg/dL, prothrombin time–international normalized ratio (PT-INR) 3.92, and activated partial thromboplastin time (APTT) 54.6 s. Abdominal ultrasonography demonstrated gallbladder wall thickening. Based on these findings, acute cholecystitis was diagnosed as TG18 Grade I according to the Tokyo Guidelines 2018.

Given the need for continuous anticoagulation for the LVAD, PTGBD was performed on hospital day 1 after administration of 4-factor prothrombin complex concentrate (Kcentra; CSL Behring, King of Prussia, PA, US) totaling 1950 IU (500 IU × 3.9 vials) to optimize coagulation status; the PT-INR decreased from 3.92 to 1.38 thereafter. Anticoagulation was managed in close collaboration with the cardiology team. Both bile and blood cultures showed no growth. Empirical antibiotic therapy with ampicillin/sulbactam (3 g intravenously, 4 times daily) was initiated and continued until POD 4.

Initial chest and abdominal radiographs were obtained to assess the position of the LVAD and the course of the driveline. The radiographs demonstrated that the LVAD pump was located in the extraperitoneal space, with the driveline running obliquely along the abdominal wall without evidence of device-related complications (**Fig. 1A** and **1B**). The patient had a continuous-flow HeartMate II LVAD. On preoperative imaging, the LVAD pump was located in an extraperitoneal (preperitoneal) abdominal wall pocket, and the driveline coursed within the abdominal wall and exited at the left upper abdomen. This information was reviewed preoperatively to facilitate incision planning and to avoid driveline injury. Contrast-enhanced CT demonstrated gallstones, gallbladder wall thickening, and pericholecystic fat stranding, findings consistent with acute cholecystitis (**Fig. 2**). Because of the high bleeding risk associated with chronic warfarin therapy and the hemodynamic vulnerability inherent to LVAD physiology, immediate surgical intervention was considered high risk. Therefore, PTGBD was performed via a transhepatic route, resulting in prompt symptomatic relief and effective infection control.

**Fig. 1:**
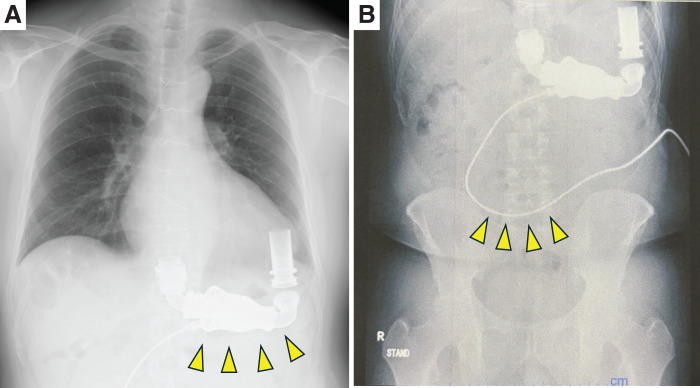
Preoperative radiographic assessment of LVAD position and driveline course. (**A**) Chest radiograph showing the LVAD pump situated in the extraperitoneal space (yellow arrowheads), with the driveline running obliquely along the right rectus abdominis muscle before exiting the left upper quadrant. (**B**) Abdominal radiograph demonstrating the overall subcutaneous course of the driveline, providing essential information for safe operative planning. The pump was positioned in an extraperitoneal (preperitoneal) abdominal wall pocket, and the driveline exited at the left upper abdomen. LVAD, left ventricular assist device

**Fig. 2:**
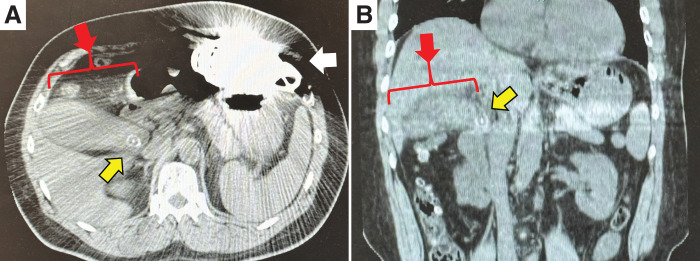
Contrast-enhanced CT findings of acute cholecystitis. (**A**) Axial and (**B**) coronal contrast-enhanced CT images showing gallbladder wall thickening and pericholecystic inflammatory changes (red arrows and brackets), with gallstones in the gallbladder neck/cystic duct region (yellow arrows). The LVAD pump-related artifact is also visible on the axial image (white arrow). LVAD, left ventricular assist device

However, the patient was in a clinical status in which donor availability could occur at any time, and the cardiology team requested definitive surgical management to ensure continuous eligibility for heart transplantation. Prolonged dependence on a PTGBD catheter was considered undesirable, as recurrent biliary infection or catheter-related complications could delay or preclude transplantation. Accordingly, the patient was referred to our department (Department of Surgery, Nagasaki University Graduate School of Biomedical Sciences) for definitive treatment. A multidisciplinary conference involving cardiology, anesthesiology, cardiovascular surgery, and hepatobiliary surgery was convened to assess operative risks and determine the safest surgical strategy.

Preoperative imaging findings, together with radiographic assessment of the LVAD and driveline anatomy, were carefully reviewed to plan a safe surgical approach. This anatomical configuration was critical for operative planning, as inadvertent injury to the driveline during abdominal entry could have resulted in catastrophic device failure.

Given the patient’s tenuous cardiac function and the risk of hemodynamic compromise from preload reduction associated with pneumoperitoneum, the anesthesiology team advised against a laparoscopic approach. Preoperative cardiac status was left ventricular ejection fraction 16%, right ventricular (RV) function severely reduced (RV fractional area change [RVFAC] 19%, tricuspid annular plane systolic excursion [TAPSE] 8 mm), pulmonary artery systolic pressure 21 mmHg, LVAD type HeartMate II, pump speed 8200 rpm, and flow 5.5 L/min. Therefore, an open cholecystectomy was selected to minimize intraoperative cardiovascular stress and avoid potential interference with LVAD physiology. Perioperative anticoagulation was carefully coordinated. The patient’s PT-INR, which typically ranged from 2.0 to 2.6 under chronic warfarin therapy, was reduced to approximately 1.4–1.5 using a heparin-bridging strategy. Intravenous heparin was continued until just before surgery and resumed promptly afterward to minimize the risk of pump thrombosis and systemic thromboembolic events. In anticipation of potential intraoperative cardiovascular instability or LVAD-related complications, the procedure was performed in collaboration with the cardiovascular surgery team. Aspirin was also temporarily withheld perioperatively and restarted at 100 mg/day on POD 2 after confirming the absence of active bleeding. The perioperative course, including anticoagulation management and serial laboratory trends, is summarized in **Fig. 3**.

**Fig. 3:**
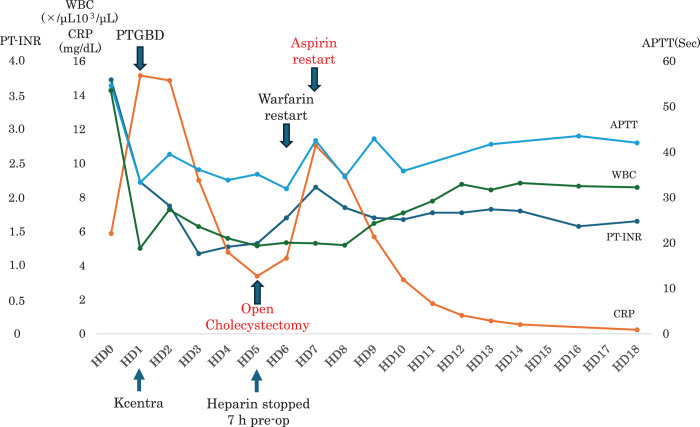
Chronological changes in inflammatory and coagulation parameters and key perioperative interventions. Serial trends of WBC count (×10^3^/µL), CRP (mg/dL), PT-INR, and APTT (s) are shown from HD 0 (admission) to discharge. PTGBD was performed on HD 1 after administration of 4F-PCC (Kcentra; total 1950 IU), resulting in a decrease in PT-INR from 3.92 to 1.38. Open cholecystectomy was performed on HD 5 (POD 0). Intravenous unfractionated heparin was discontinued 7 h before surgery. Warfarin was restarted on HD 6 at 2.5 mg, and aspirin was restarted at 100 mg/day on HD 7 (POD 2) after confirming the absence of active bleeding. 4F-PCC, 4-factor prothrombin complex concentrate; APTT, activated partial thromboplastin time; CRP, C-reactive protein; HD, hospital day; Kcentra, CSL Behring, King of Prussia, PA, USA; PTGBD, percutaneous transhepatic gallbladder drainage; PT-INR, prothrombin time–international normalized ratio; WBC, white blood cell

The operation was performed 4 days after PTGBD. Immediately before skin incision, ultrasonography was used to delineate the subcutaneous course of the LVAD driveline, and its trajectory was carefully marked on the skin with indelible ink (**Fig. 4**). The driveline connection site was also identified and marked to avoid inadvertent injury during abdominal entry. Based on these landmarks, a right subcostal incision was planned and drawn so that the skin incision would not intersect the driveline course while still providing optimal exposure to the gallbladder. After reconfirming the driveline position and the location of the PTGBD catheter, the right subcostal incision was made and the abdomen was entered safely. Representative photographs of the incision planning and wound closure are shown in **Fig. 5**.

**Fig. 4:**
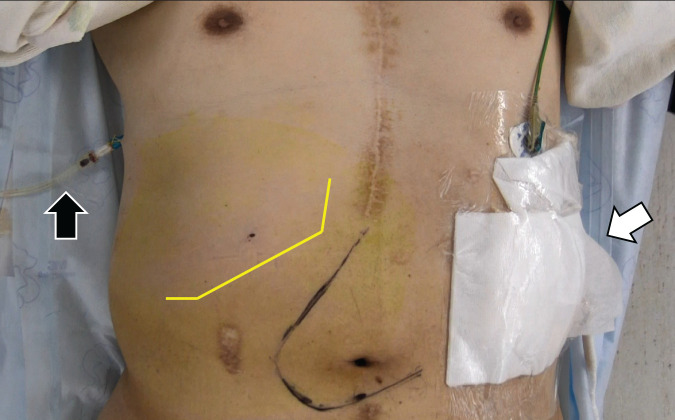
Preoperative marking of the LVAD driveline and planned skin incision.US was used to confirm the course of the LVAD driveline, which was marked on the skin with a black line. The driveline’s subcutaneous connection site is indicated by a white arrow, and the existing PTGBD catheter is shown by a black arrow. The yellow line represents the planned right subcostal skin incision, drawn to avoid the driveline and allow safe operative entry. LVAD, left ventricular assist device; PTGBD, Percutaneous transhepatic gallbladder drainage

**Fig. 5:**
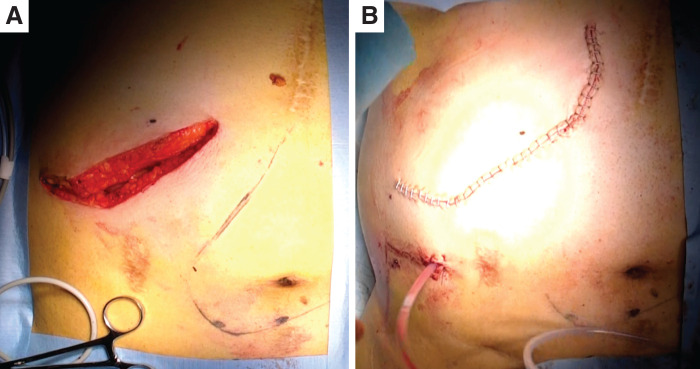
Representative intraoperative photographs. (**A**) Skin incision initially planned to avoid the driveline, which was subsequently extended with an upper midline abdominal incision. (**B**) Wound appearance after closure, demonstrating adequate separation from the driveline exit site.

Dense adhesions and inflammatory changes were present around the gallbladder. Careful dissection revealed a thickened gallbladder with multiple embedded gallstones. The cystic artery and cystic duct were clearly identified, ligated, and divided in a standard fashion. The total operative time was 2 h and 14 min, and the estimated blood loss was 516 mL, necessitating transfusion of 280 mL of packed red blood cells. No injury to the LVAD pump, outflow graft, or driveline occurred. Postoperatively, the patient was admitted to the ICU for close hemodynamic and anticoagulation management.

Pathological examination demonstrated chronic cholecystitis with cholelithiasis, and no malignant findings were identified. Postoperatively, the patient remained hemodynamically stable, and there was no uncontrollable bleeding and no bleeding-related complications despite the challenges of perioperative anticoagulation management. The abdominal drain was removed on POD 2, and his subsequent clinical course was uneventful. He was discharged home in good condition on POD 15. Four months after the cholecystectomy, he successfully underwent orthotopic heart transplantation, resulting in an excellent overall clinical outcome, including successful heart transplantation.

## DISCUSSION

This case illustrates the significant challenges associated with the management of acute cholecystitis in patients supported by an LVAD. Safe perioperative care required close collaboration among cardiology, cardiovascular surgery, anesthesiology, and hepatobiliary surgery. In particular, careful anticoagulation management and detailed operative planning, with special attention to driveline anatomy and surgical approach, were essential to achieving a favorable outcome. Importantly, definitive treatment of biliary infection before heart transplantation was critical in this case, as unresolved or recurrent infection could have jeopardized transplant eligibility.

With the expanding indications and improved long-term outcomes of LVAD therapy, the number of patients living for extended periods with mechanical circulatory support has markedly increased.^4)^ Recent series have reported that approximately 10%–15% of LVAD recipients undergo at least 1 noncardiac operation during support, with abdominal emergencies such as acute cholecystitis, appendicitis, and gastrointestinal bleeding among the most frequent indications.^5–7)^ These patients present unique perioperative challenges due to 3 interrelated factors: (1) the need for continuous anticoagulation to prevent pump thrombosis, (2) the altered hemodynamic physiology associated with continuous-flow support, and (3) the presence of device hardware, including the pump pocket and driveline.^5)^ Consequently, standard perioperative management alone is often insufficient, and multidisciplinary planning is essential to optimize operative timing, surgical approach, and anticoagulation management.

Among noncardiac surgical problems, acute cholecystitis is one of the most frequent abdominal indications in LVAD-supported patients and warrants special attention because of its strong association with infection-related morbidity. Biliary infection in this population can rapidly progress to bacteremia or driveline/pocket infection, directly threatening LVAD integrity and heart transplant candidacy.^8)^ For hemodynamically fragile patients, PTGBD is commonly selected as the initial strategy because it provides reliable infection control with minimal physiological stress.^3,9)^ Although early cholecystectomy is generally recommended for acute cholecystitis, decision-making in LVAD-supported patients is complex due to continuous anticoagulation and device-specific hemodynamic vulnerability. In this case, despite TG18 Grade I acute cholecystitis, urgent cholecystectomy was considered high risk because of LVAD support, chronic anticoagulation, and severely reduced right ventricular function. PTGBD therefore served as a bridge to definitive surgery by providing prompt source control while enabling optimization of anticoagulation and multidisciplinary perioperative planning. However, definitive cholecystectomy should not be excessively delayed, as prolonged catheter placement increases the risk of recurrent infection and tube-related complications. Previous reports consistently demonstrate that emergent abdominal operations in LVAD recipients carry significantly higher morbidity and mortality, whereas elective surgery after adequate stabilization results in markedly better outcomes. This supports the staged approach used in the present case, in which PTGBD followed by early elective open cholecystectomy resulted in an uneventful recovery and preserved transplant eligibility.^1,7)^ Previously reported LVAD-supported patients undergoing cholecystectomy, including staged cases (PTGBD followed by cholecystectomy) and the present case, are summarized in **Table 1**.^1–3,8,10–14)^

**Table 1:** Previously reported LVAD-supported patients undergoing cholecystectomy, including laparoscopic and open approaches, and the present case

Author(s)	Year	Number of cases	Diagnosis (number of cases)	Approach (number of cases)	Conversion (number of cases)	PTGBD before surgery	Postoperative complications (number of cases)
Kartha et al.^10)^	2008	1	Cholelithiasis	Laparoscopic	NR	No	No
Amir et al.^11)^	2012	1	Cholecystitis and pancreatitis	Laparoscopic	NR	No	No
Naitoh et al.^8)^	2013	1	Cholecystitis	Laparoscopic	NR	No	No
Ashfaq et al.^12)^	2016	4	Cholecystitis (3), gallstone pancreatitis with symptomaticcholelithiasis (1)	Laparoscopic	No	No	Acute kidney injury (1)
Vigneswaran et al.^2)^	2019	6	Cholelithiasis (4), cholecystitis (1), gallstone pancreatitis (1)	Laparoscopic	No	No	No
Suresh et al.^13)^	2019	5	Cholelithiasis (4), cholecystitis (1)	Laparoscopic	NR	No	Abdominal wall hematoma (1)
Takagi et al.^14)^	2019	1	Abdominal hemorrhage	Laparoscopic	No	No	No
Zilbermints et al.^1)^	2020	4	Cholecystitis (2), gangrenous cholecystectitis (2)	Laparoscopic (2)Open (2)	Yes (2)	No	Lap group: transfusion (1), open group: death (2)
Hanaki et al.^3)^	2022	1	Cholecystitis	Laparoscopic (elective after PTGBD)	No	Yes	No
Our case		1	Cholecystitis	Open	NA	Yes	No

This table summarizes number of cases, diagnosis/indication, surgical approach and conversion, whether PTGBD was performed before surgery, and postoperative complications/outcomes. In the PTGBD before surgery column, “Yes” indicates that PTGBD was performed before cholecystectomy, whereas “No” indicates that it was not performed. In the postoperative complications column, “Yes” indicates that postoperative complications occurred, whereas “No” indicates that no postoperative complications occurred.

LVAD, left ventricular assist device; NA, not applicable; NR, not reported; PTGBD, percutaneous transhepatic gallbladder drainage

The choice of surgical approach must be individualized. Laparoscopic cholecystectomy has been reported as feasible in selected stable LVAD patients when appropriate pneumoperitoneum is used and meticulous preoperative mapping of the driveline is performed.^3,10,14)^ In the present case, a laparoscopic approach was initially considered. However, pneumoperitoneum has been reported to affect preload and induce significant hemodynamic changes in patients with LVAD support.^15)^ Although laparoscopic cholecystectomy has been reported in selected LVAD-supported patients, pneumoperitoneum and patient positioning may reduce venous return and decrease LVAD preload, while increasing right ventricular afterload, potentially leading to hemodynamic instability. In our patient, severely reduced right ventricular function (RVFAC 19%, TAPSE 8 mm) and the need to avoid any perioperative cardiopulmonary deterioration that could compromise transplant eligibility supported the selection of an open approach after multidisciplinary discussion. After thorough preoperative discussions with the anesthesiology and cardiovascular surgery teams, we considered that pneumoperitoneum could increase right ventricular afterload, potentially leading to circulatory failure, and might also interfere with LVAD inflow and outflow. In addition, concerns regarding the management of intraoperative bleeding under anticoagulation favored an open approach. Consequently, open cholecystectomy was selected as the safer operative strategy. Preoperative biliary imaging such as magnetic resonance cholangiopancreatography or cholangiography through the PTGBD catheter was not obtained in this case because the patient showed clinical improvement after PTGBD and there was no imaging evidence suggesting choledocholithiasis or biliary obstruction. In addition, early definitive surgery was prioritized to eliminate the biliary infectious source and maintain heart transplant eligibility. Therefore, particular care was taken intraoperatively to avoid bile duct injury, including meticulous dissection of Calot’s triangle and confirmation of the critical view of safety before clipping and dividing the cystic duct and artery.

Preoperative imaging to delineate the anatomy of the LVAD and its driveline is essential, as inadvertent injury can result in catastrophic device malfunction or severe infection. The course of the driveline is typically evaluated using abdominal radiography or CT,^2)^ which allows precise preoperative mapping and helps guide safe port placement or incision planning. In our case, US-guided surface marking of the driveline provided additional real-time information that was valuable not only for determining the optimal incision site but also for minimizing mechanical stress on the driveline during wound retraction and surgical manipulation.

Perioperative anticoagulation management in LVAD recipients undergoing noncardiac surgery is a critical determinant of procedural safety. This management requires partial reversal of warfarin with conversion to intravenous heparin, close monitoring of coagulation parameters (including PT-INR and APTT), and appropriate adjustment of antiplatelet therapy to balance the competing risks of bleeding and pump thrombosis. In the present case, aspirin 100 mg/day was temporarily withheld perioperatively and restarted on POD 2 after confirming the absence of active bleeding. Previous studies have reported preoperative PT-INR levels of approximately 1.7 achieved through partial reversal of warfarin.^2)^ Bridging with intravenous heparin is typically maintained until just before surgery, followed by early postoperative resumption of anticoagulation.^10,16,17)^ Our patient was managed according to this strategy and experienced no uncontrollable bleeding, no bleeding-related complications, and no thromboembolic complications.

Overall, the physiological and hematological characteristics inherent to LVAD support necessitate an individualized perioperative strategy that integrates tailored anticoagulation management, vigilant device monitoring, and careful selection of surgical technique. As clinical experience continues to accumulate, insights from cases such as the present one will contribute to refining standardized algorithms for the safe surgical management of LVAD recipients, particularly those awaiting heart transplantation.

## CONCLUSIONS

Acute cholecystitis in patients supported by an LVAD can be safely managed using a staged strategy consisting of early infection control followed by elective cholecystectomy. Successful outcomes require strict multidisciplinary collaboration, meticulous perioperative anticoagulation management, vigilant device monitoring, and precise preoperative assessment of driveline anatomy to prevent hemodynamic instability and device-related injury. Prompt eradication of biliary infection is essential to preserve heart transplant eligibility. As the population of patients living with long-term LVAD support continues to grow, individualized perioperative strategies will be increasingly important to ensure safe and effective surgical care in this high-risk group.

## DECLARATIONS

### Availability of data and materials

The data supporting the findings of this study are available from the corresponding author upon reasonable request.
